# The Intersection of Physical Inactivity, Dementia, and Gender: A Population-Based Study of All-Cause Mortality in Middle-Aged and Older Adults in Korea

**DOI:** 10.3390/medicina62040628

**Published:** 2026-03-26

**Authors:** Gyeong-Min Lee, Yu-Seung Lee, Jae-Hyun Kim

**Affiliations:** 1 Research Institute for Healthcare Policy, Dankook University, Cheonan 31116, Republic of Korea; lgm910212@dankook.ac.kr (G.-M.L.); yslee_ph@dankook.ac.kr (Y.-S.L.); 2 Department of Health Administration, College of Public Health Sciences, Dankook University, Cheonan 31116, Republic of Korea

**Keywords:** dementia, physical activity, all-cause mortality, aging, gender differences

## Abstract

*Background and Objectives*: Physical activity and dementia are important determinants of mortality in aging populations, yet their joint association remains insufficiently understood. This study examined the combined association of physical activity and dementia with all-cause mortality in a nationally representative cohort of Korean adults. *Materials and Methods*: We used data from the Korean Longitudinal Study of Aging (KLoSA), with baseline in 2018 and follow-up through 2022. A total of 6935 participants were included. Physical activity was categorized based on weekly exercise duration using World Health Organization recommendations (Inactive <150 min/week, and ≥150 min/week). Dementia was defined by self-reported physician diagnosis. Cox proportional hazards models were used to estimate hazard ratios (HRs) for mortality, adjusting for relevant covariates. Sex-stratified analyses were conducted as sensitivity analyses. *Results*: During follow-up, 597 deaths occurred. Participants with both dementia and inactivity had the highest mortality risk. Sufficient physical activity (≥150 min/week) was associated with lower mortality risk overall. In sex-stratified analyses, the protective association of sufficient physical activity appeared more evident among women; however, subgroup findings should be interpreted cautiously due to limited events. *Conclusions*: Physical inactivity and dementia were jointly associated with increased mortality risk. Maintaining adequate physical activity may be particularly important in cognitively vulnerable populations, although further research is needed to confirm subgroup-specific patterns.

## 1. Introduction

Population aging has emerged as one of the most significant global public health challenges of the twenty-first century [1]. According to recent estimates, the number of people aged 65 years and older worldwide is projected to double between 2020 and 2050, reaching more than 1.5 billion individuals [2]. Alongside this demographic shift, the global burden of dementia has increased rapidly, with over 55 million people currently living with dementia worldwide and nearly 10 million new cases occurring each year [3]. Dementia is strongly associated with premature mortality, functional decline, and substantial healthcare costs, making it a major contributor to the global burden of disease among older adults [4].

Korea is experiencing population aging at an unprecedented pace. The proportion of adults aged 65 years and older has increased sharply over the past two decades, transitioning the country into an aged society and approaching super-aged status [5]. Parallel to this trend, the prevalence of dementia among older Koreans has risen steadily, with recent national estimates indicating that approximately one in ten adults aged 65 years or older is affected [6]. Projections suggest that the absolute number of individuals with dementia will continue to grow substantially over the coming decades [7]. These demographic and epidemiological changes have heightened concerns regarding excess mortality, healthcare utilization, and quality of life among older adults with cognitive impairment, underscoring the need for evidence-based strategies to mitigate adverse outcomes [8].

Physical activity has long been recognized as a modifiable health behavior associated with reduced mortality and improved functional outcomes in the general population [9]. A substantial body of epidemiological research has demonstrated that regular physical activity is linked to lower risks of cardiovascular disease, metabolic disorders, and all-cause mortality [10]. In older adults, even moderate levels of physical activity have been associated with survival benefits, suggesting that exercise may play a critical role in promoting healthy aging [11]. Consequently, physical activity has been widely emphasized in public health guidelines as a key intervention to reduce mortality risk across the life course [12].

In recent years, growing attention has been directed toward the role of physical activity in populations with dementia or cognitive impairment [13]. Several studies have suggested that exercise may slow cognitive decline, improve physical functioning, and enhance quality of life among individuals with dementia [14,15]. However, evidence regarding the association between exercise and mortality in this population remains limited and inconsistent. Some studies have reported protective associations between physical activity and survival among individuals with cognitive impairment, while others have found attenuated or non-significant effects, particularly in advanced stages of dementia [16,17].

Moreover, many existing studies have examined exercise or dementia in isolation, without adequately considering their combined effects on mortality [18]. Research focusing on the joint relationship between physical activity levels and dementia status remains relatively sparse, and few studies have directly compared mortality risks across groups defined by both exercise behavior and dementia. This limitation is particularly evident in population-based studies with sufficient follow-up to evaluate survival outcomes [18]. In addition, previous investigations have often relied on small clinical samples, short follow-up periods, or limited adjustment for social and health-related confounders, restricting the generalizability of their findings. While Park et al. [19] demonstrated population-level associations between physical activity and mortality, the present study extends this literature by examining how physical activity operates within the everyday social context of community-dwelling older adults with dementia [19].

Another important gap in the existing literature is the insufficient exploration of gender differences in the association between exercise, dementia, and mortality. Gender-related differences in health behaviors, disease progression, and social support structures may modify the impact of physical activity on survival among individuals with and without dementia [20,21]. Nevertheless, evidence on whether the mortality benefits of exercise differ by gender in the context of dementia remains scarce, particularly in nationally representative longitudinal samples.

Given these gaps, the present study aimed to examine the joint associations incorporating physical inactivity, dementia status, social contact, and gender, rather than merely estimating independent associations. By jointly classifying individuals according to exercise duration and dementia status, this study sought to clarify how varying levels of physical activity are associated with mortality risk among those with and without dementia. In addition, gender-stratified analyses were conducted to assess potential differences in these associations between men and women. By integrating behavioral and cognitive factors within a joint exposure framework, this study provides population-based evidence on how modifiable health behaviors and cognitive aging interact to shape mortality risk. Although social contact was not modeled as an interaction term, it was included as a key contextual covariate to capture the social embeddedness of physical activity in later life. Given the limited number of dementia cases, the present study focused on interpretative integration rather than formal interaction testing.

We hypothesized that lower levels of exercise would be associated with increased all-cause mortality, and that this association would be more pronounced among individuals with dementia. We further hypothesized that the joint association between exercise and dementia with mortality would differ by gender.

## 2. Methods

### 2.1. Study Design and Study Population

This study used data from the Korean Longitudinal Study of Aging (KLoSA), a nationally representative panel survey of community-dwelling adults in Korea. The present analysis used 2018 as the baseline and followed participants through 2022. A total of 6935 individuals were included in the analysis.

Dementia status was determined based on self-reported physician diagnosis obtained from the survey questionnaire. Because the measure relied on self-reported information, it may be subject to reporting bias. Yielding 94 individuals with dementia and 6841 individuals without dementia in the analytic sample. The KLoSA employs a multistage stratified probability sampling design, and all analyses accounted for sampling weights to ensure national representativeness.

### 2.2. Measures

#### 2.2.1. Outcome Variable

The primary outcome of this study was all-cause mortality. Mortality status was identified based on follow-up information available in the KLoSA dataset and was coded as a binary variable, with death coded as 1 and survival as 0. Survival time was calculated from the baseline survey year (2018) to the year of death or the end of follow-up, whichever occurred first.

#### 2.2.2. Independent Variables

The primary independent variable was the joint status of physical activity and dementia. Weekly physical activity was calculated by multiplying the self-reported frequency of exercise sessions by the average duration per session, resulting in total minutes of exercise per week. Based on this measure, Physical activity was dichotomized into inactive (<150 min/week) and sufficient (≥150 min/week), based on WHO recommendations. Participants with less than 150 min of weekly activity were considered not meeting recommended activity levels. This classification enhances clinical relevance and aligns with widely accepted public health guidelines [22,23]. Dementia status was defined based on self-reported physician diagnosis in the survey. Participants who reported a diagnosis of dementia were classified as having dementia, whereas those with normal cognition or mild cognitive impairment were included in the non-dementia group.

To examine their combined association with mortality, physical activity and dementia status were jointly classified into mutually exclusive categories. Baseline characteristics of the study population are presented in Table 1. In the primary analysis (Table 2), participants with inactive and no dementia were used as the reference group to provide a clinically intuitive comparison across risk strata.

#### 2.2.3. Covariates

Several sociodemographic, health-related, and social variables were included as covariates. These variables were selected based on prior literature and data availability. Depression was assessed using a survey item asking about physician-diagnosed depression and current antidepressant use and was coded as a binary variable (yes vs. no). Gender was categorized as male or female, and age was grouped into ≤64, 65–74, and ≥75 years. Educational attainment was classified as elementary school or less, middle school, high school, and college or higher. Marital status was categorized as married, divorced or widowed, or single.

Region of residence was classified as metropolitan, urban, or rural. Self-rated health was measured using a single-item question and categorized as good, fair, or poor. Social contact frequency was assessed based on the reported frequency of contact with family members or acquaintances and categorized into five levels: everyday, two to three times per week, one to two times per month, five to six times per year, and rarely. All covariates were measured at baseline and treated as fixed variables in the analyses.

### 2.3. Statistical Analysis

Baseline characteristics were summarized according to mortality status using weighted frequencies and percentages, and group differences were assessed using chi-square tests. Cox proportional hazards regression models were used to estimate hazard ratios (HRs) and 95% confidence intervals (CIs) for mortality.

The primary analysis examined the joint association of physical activity and dementia using combined exposure groups. To improve interpretability and reduce instability due to sparse data, exposure categories were simplified and structured to reflect clinically meaningful contrasts.

Sex-stratified analyses were additionally performed as sensitivity analyses, in which physical activity and dementia were modeled separately within each sex.

All models were adjusted for relevant covariates. The proportional hazards assumption was assessed using graphical methods and time-dependent covariates, and no major violations were detected. Given the limited number of events in some subgroups, results from these categories were interpreted cautiously. All analyses were conducted using SAS version 9.4 (SAS Institute Inc., Cary, NC, USA), and statistical significance was defined as a two-sided *p* value < 0.05.

## 3. Results

A total of 6935 participants were included in the analysis, among whom 597 deaths occurred during follow-up. Baseline characteristics according to mortality status are presented in Table 1. Physical activity level and dementia status both differed significantly by mortality status. Participants with little physical activity (<150 min/week) had the highest proportion of deaths (10.22%), whereas those with sufficient physical activity (≥150 min/week) had the lowest mortality proportion (4.79%) (*p* < 0.001). Dementia was also strongly associated with mortality, with 50.0% of participants with dementia dying during follow-up compared with 8.03% among those without dementia (*p* < 0.001). Because some joint physical activity–dementia categories contained very small cell counts, the distribution of mortality across these groups was evaluated using the Fisher–Freeman–Halton exact test, which showed a significant difference (*p* < 0.001).

**Table 1:** Baseline characteristics of the study population by mortality status (*N* (%)).

Characteristics	Total (*N* = 6935)	Mortality: Yes (*N* = 592)	Mortality: No (*N* = 6343)	*p*-Value
Exercise level (min/week)				<0.0001
Inactive (<150)	5160 (74.4)	507 (9.8)	4653 (90.2)	
Sufficient exercise (≥150)	1775 (25.6)	85 (4.8)	1690 (95.2)	
Dementia				<0.0001
Yes	94 (1.4)	47 (50.0)	47 (50.0)	
No	6841 (98.6)	545 (8.0)	6296 (92.0)	
Depression				<0.0001
Yes	248 (3.6)	60 (24.2)	188 (75.8)	
No	6687 (96.4)	532 (8.0)	6155 (92.0)	
Gender				0.0001
Male	2933 (42.3)	294 (10.0)	2639 (90.0)	
Female	4002 (57.7)	298 (7.5)	3704 (92.5)	
Age (years)				<0.0001
≤64	2587 (37.3)	38 (1.5)	2549 (98.5)	
65–74	2035 (29.3)	88 (4.3)	1947 (95.7)	
≥75	2313 (33.4)	466 (20.1)	1847 (79.9)	
Education level				<0.0001
≤Elementary school	2675 (38.6)	380 (14.2)	2295 (85.8)	
Middle school	1153 (16.6)	83 (7.2)	1070 (92.8)	
High school	2226 (32.1)	94 (4.2)	2132 (95.8)	
≥College	881 (12.7)	35 (4.0)	846 (96.0)	
Marital status				<0.0001
Married	5229 (75.4)	341 (6.5)	4888 (93.5)	
Divorced/Widowed	1649 (23.8)	247 (15.0)	1402 (85.0)	
Single	57 (0.8)	4 (7.0)	53 (93.0)	
Region				<0.0001
Metropolitan	2924 (42.2)	216 (7.4)	2708 (92.6)	
Urban	2291 (33.0)	183 (8.0)	2108 (92.0)	
Rural	1720 (24.8)	193 (11.2)	1527 (88.8)	
Self-rated health (SRH)				<0.0001
Good	2047 (29.5)	50 (2.4)	1997 (97.6)	
Moderate	3052 (44.0)	192 (6.3)	2860 (93.7)	
Poor	1836 (26.5)	350 (19.1)	1486 (80.9)	
Contact frequency				<0.0001
Everyday	1383 (19.9)	118 (8.5)	1265 (91.5)	
2–3 times/week	2440 (35.2)	166 (6.8)	2274 (93.2)	
1–2 times/month	1688 (24.3)	88 (5.2)	1600 (94.8)	
5–6 times/year	712 (10.3)	53 (7.4)	659 (92.6)	
Rarely	712 (10.3)	167 (23.5)	545 (76.5)	

**Note.** Values are presented as number (%). *p*-values were calculated using chi-square tests, or the Fisher–Freeman–Halton exact test when expected cell counts were less than five. Physical activity was categorized as inactive (<150 min/week) and sufficient (≥150 min/week). Dementia status was identified based on self-reported physician diagnosis.

Table 2 presents the results of the Cox proportional hazards models examining physical activity and dementia (modeled simultaneously) with all-cause mortality. Physical activity was dichotomized into inactive and sufficient categories; therefore, no intermediate category was included in the model.

In the fully adjusted model, participants with dementia who did not engage in physical activity had the highest mortality risk (adjusted HR = 2.301, 95% CI: 1.669–3.172; *p* < 0.001), compared with the reference group (inactive and no dementia). In contrast, individuals without dementia who engaged in sufficient physical activity (≥150 min/week) showed a significantly lower mortality risk (adjusted HR = 0.722, 95% CI: 0.564–0.925; *p* = 0.01).

The subgroup of participants with both sufficient physical activity and dementia did not show a statistically significant association with mortality (adjusted HR = 1.050, 95% CI: 0.146–7.563; *p* = 0.962), likely reflecting the extremely small number of participants in this category.

**Table 2:** Association of physical activity and dementia with all-cause mortality (Cox proportional hazards model).

Variable	Adjusted HR (95% CI)	*p*-Value
Exercise level (ref: Inactive)		
Sufficient Exercise (≥150)	0.723 (0.568–0.921)	0.0087
Dementia (ref: No)		
Yes	2.257 (1.641–3.104)	<0.0001
Depression (ref: No)		
Yes	1.724 (1.305–2.279)	<0.0001
Gender (ref: Female)		
Male	2.266 (1.866–2.753)	<0.0001
Age group (ref: ≤64 years)		
65–74 years	2.151 (1.452–3.186)	<0.0001
≥75 years	7.603 (5.278–10.952)	<0.0001
Education level (ref: ≥College)		
Elementary school	1.480 (1.018–2.152)	0.040
Middle school	1.448 (0.968–2.164)	0.071
High school	1.153 (0.781–1.704)	0.474
Marital status (ref: Married)		
Divorced/Widowed	1.545 (1.274–1.874)	<0.0001
Single	1.363 (0.500–3.716)	0.545
Region (ref: Metropolitan)		
Urban	0.955 (0.782–1.166)	0.649
Rural	1.093 (0.892–1.338)	0.392
Self-rated health (ref: Good)		
Moderate	1.552 (1.131–2.131)	0.006
Poor	2.655 (1.928–3.656)	<0.0001
Social contact frequency (ref: Everyday)		
2–3 times/week	1.076 (0.848–1.365)	0.547
1–2 times/month	1.113 (0.837–1.480)	0.463
5–6 times/year	1.265 (0.906–1.765)	0.167
Rarely	1.765 (1.371–2.272)	<0.0001

**Figure 1:**
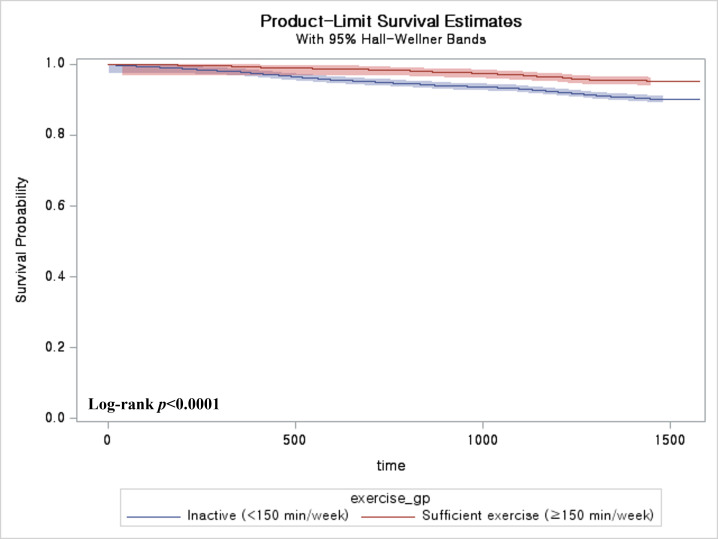
Kaplan–Meier curves for all-cause mortality according to physical activity level. **Note.** Participants were classified as inactive (<150 min/week) or sufficiently active (≥150 min/week). Survival probability was higher in the sufficiently active group compared with the inactive group (log-rank *p* < 0.0001).

Kaplan–Meier curves for physical activity alone are presented in Figure 1. Participants engaging in sufficient physical activity demonstrated consistently higher survival probabilities compared with those reporting inactivity, with an intermediate pattern observed among those with inactivity. These patterns were consistent with the results of the multivariable analyses.

Kaplan–Meier curves for joint categories are presented in Supplementary Figure S1. Survival probabilities differed markedly across groups, with the lowest survival observed among participants with both dementia and inactivity. In contrast, participants without dementia who engaged in sufficient physical activity (≥150 min/week) showed the highest survival probability throughout the follow-up period. The separation of the survival curves was evident early and persisted over time, supporting the findings from the Cox regression analysis.

Sex-stratified analyses are presented in Table 3, where the associations ofphysical activity and dementia with mortality were examined separately by sex.

Among women, sufficient physical activity (≥150 min/week) was associated with a significantly lower risk of mortality (adjusted HR = 0.513, 95% CI: 0.324–0.813; *p* = 0.0045), whereas no statistically significant association was observed among men. Dementia was associated with increased mortality in both sexes, with a stronger association observed in women (adjusted HR = 2.731, 95% CI: 1.844–4.045; *p* < 0.001) than in men, although the estimate in men did not reach statistical significance.

Other covariates, including older age and poor self-rated health, were consistently associated with increased mortality in both sexes. Infrequent social contact was significantly associated with mortality among women but showed a weaker and less consistent pattern among men.

Given the limited number of events in some subgroups and the absence of formal interaction testing, these sex-specific findings should be interpreted cautiously.

Due to sparse events in some joint categories, the combined physical activity–dementia model was presented as supplementary analysis. Supplementary analyses examining physical activity and dementia separately are presented in Supplementary Tables and Figures. These analyses showed patterns generally consistent with the main findings.

## 4. Discussion

This study examined the joint association between physical activity and dementia with all-cause mortality among community-dwelling adults in Korea. Using nationally representative longitudinal data, we found that individuals with both dementia and inactivity exhibited the highest mortality risk. In contrast, sufficient physical activity (≥150 min/week) was associated with lower mortality risk, particularly among individuals without dementia. These findings suggest that physical inactivity and dementia were jointly associated with increased mortality risk, rather than acting as isolated risk factors.

Our findings are consistent with a growing body of literature demonstrating that physical activity is associated with reduced all-cause mortality in older adults [16]. Previous large-scale population-based studies have also reported that physical activity is associated with improved survival among individuals with dementia [19,24]. These findings highlight the potential importance of maintaining physical activity even in cognitively impaired populations.

While such large administrative datasets are well suited to identifying general epidemiological trends, they are inherently limited in their ability to capture the social and lived contexts in which physical activity occurs. The present study extends prior work by focusing on community-dwelling older adults and incorporating social contact as a key contextual factor alongside physical activity and dementia [25]. By doing so, our findings suggest that the association between physical activity and mortality among individuals with dementia may not be attributable solely to physiological mechanisms, but may also reflect broader social processes embedded in everyday life [26].

An important conceptual contribution of this study lies in the qualitative reinterpretation of “physical inactivity,” particularly among individuals with dementia. In older populations, and especially among those with cognitive impairment, reporting “inactive” may not simply indicate the absence of leisure-time physical activity. Rather, it may signal functional decline that precludes even low-intensity activities such as walking, household tasks, or routine outdoor movement [27]. This interpretation is particularly relevant in the context of dementia, where declining cognitive function is often accompanied by reduced mobility, frailty, and loss of independence [28,29].

From this perspective, physical inactivity may serve as a proxy indicator of accumulated vulnerability, may reflect the combined effects of physical frailty, cognitive impairment, and reduced social engagement. However, causal inference cannot be established due to the observational study design. The substantially elevated mortality risk observed among individuals with both dementia and inactivity supports this interpretation and suggests that inactivity captures a critical threshold beyond which survival is substantially compromised [12].

The findings related to social contact further support the notion that physical activity operates within a broader social context. Infrequent social contact was associated with increased mortality, particularly among women, and the coexistence of physical inactivity and limited social interaction may compound mortality risk. Although the present analysis could not fully disentangle whether physical activity occurred alone or in social settings, social contact may act as a proxy for socially embedded physical activity, such as participation in group exercise programs, community-based activities, or routine interactions involving movement [30].

This perspective raises the possibility that the association between physical activity and mortality among individuals with dementia may be partially influenced by social mechanisms, including reduced social isolation, enhanced emotional support, and greater engagement in daily routines. Thus, physical activity in later life may represent not only a biological factor but also a socially embedded behavior that supports functional capacity and social connectedness [31].

Gender-stratified analyses suggested potential differences between men and women in the association between physical inactivity, dementia, and mortality. Previous studies have reported that gender differences in physical activity patterns and social participation may influence health outcomes in later life [32]. However, because the formal interaction test between gender and the exposure categories was not statistically significant, and some subgroup estimates were based on limited events, these findings should be interpreted cautiously.

In the Korean sociocultural context, older women have traditionally borne primary responsibility for household labor and caregiving throughout their lives [33]. For these women, the absence of physical activity may indicate not merely a lack of structured exercise, but a decline in the ability to perform daily activities [34]. In contrast, physical inactivity among older men may more often reflect reduced participation in leisure-time exercise rather than complete functional dependence [35]. Consequently, “inactive” may reflect different underlying conditions across genders.

Moreover, gendered caregiving structures may further contribute to these differences. Older men with dementia are more likely to receive spousal care, which may help maintain routine activity and access to healthcare [36]. Older women with dementia, by contrast, are more likely to be widowed or live alone, potentially increasing vulnerability to functional decline and mortality [36,37]. The associations observed for marital status and social contact in this study are consistent with this interpretation.

The present study also highlights the importance of appropriate methodological approaches when examining mortality in small or highly stratified subgroups. Some combinations of physical activity and dementia status were characterized by sparse events, resulting in unstable estimates. To address this issue, simplified exposure classifications were applied, and findings were interpreted conservatively. Nevertheless, because certain subgroups were extremely small, the corresponding estimates should be interpreted with caution.

These findings have important implications for public health and dementia care policy. First, maintaining physical activity, even at modest levels, may be important for survival among older adults with dementia [10,11,38]. Interventions aimed at preventing complete physical inactivity, rather than achieving high-intensity exercise, may be particularly relevant in this population [9,12]. Second, the observed gender differences suggest the need for approaches that consider differences in social roles, caregiving structures, and vulnerability across the life course [33,34,35,36,37].

Programs that integrate physical activity with social engagement, such as group-based or community-based activities, may offer complementary benefits by addressing both biological and social dimensions of health [30,31,32]. In rapidly aging societies such as Korea, expanding access to such programs for cognitively vulnerable older adults may help reduce excess mortality [5,16]. In this context, social contact may be interpreted as part of a broader behavioral and social framework within which physical activity occurs [30].

An additional issue concerns the reversal of the depression coefficient in the sex-stratified models. While depression was associated with increased mortality in the pooled model, the sex-stratified estimates suggested a lower hazard ratio. We re-examined the coding of the depression variable and confirmed that no coding errors were present. This discrepancy is likely attributable to residual confounding and instability in stratified estimates, particularly given the relatively small number of events. Therefore, these findings should be interpreted with caution.

### Limitations

Several limitations should be acknowledged. Dementia status and physical activity were based on self-reported data and may be subject to misclassification. Physical activity was measured at a single time point, limiting assessment of changes over time. The study period overlapped with the COVID-19 pandemic, which may have influenced physical activity and social contact patterns.

The number of participants with dementia was relatively small, and some subgroup analyses included very few events. In particular, only a small number of participants with dementia engaged in sufficient physical activity, resulting in unstable estimates. Therefore, these findings should be interpreted as preliminary.

## 5. Conclusions

In conclusion, physical inactivity and dementia were jointly associated with increased mortality risk among older adults. These findings suggest that maintaining physical activity may be important even among individuals with cognitive impairment. By situating physical activity within a broader social and functional context, this study highlights the potential role of socially embedded activity in supporting healthy aging.

## Figures and Tables

**Table 3:** Gender-stratified sensitivity analysis of the association between exercise and dementia status and all-cause mortality.

Variable	Male		Female	
	Adjusted HR (95% CI)	*p*-Value	Adjusted HR (95% CI)	*p*-Value
Exercise level (ref: Inactive)				
Sufficient Exercise (≥150)	0.846 (0.628–1.140)	0.2726	0.513 (0.324–0.813)	0.0045
Dementia (ref: No)				
Yes	1.481 (0.849–2.582)	0.1662	2.731 (1.844–4.045)	<0.0001
Depression (ref: No)				
Yes	2.046 (1.345–3.113)	0.0008	1.674 (1.146–2.444)	0.0077
Age group (ref: ≤64 years)				
65–74 years	1.848 (1.140–2.994)	0.0126	2.585 (1.295–5.162)	0.0071
≥75 years	6.324 (4.061–9.848)	<0.0001	9.067 (4.684–17.551)	<0.0001
Education level (ref: ≥College)				
≤Elementary school	1.539 (1.012–2.342)	0.0439	0.948 (0.376–2.391)	0.9097
Middle school	1.694 (1.081–2.657)	0.0216	0.811 (0.305–2.160)	0.6754
High school	1.255 (0.819–1.923)	0.2973	0.702 (0.260–1.895)	0.4851
Marital status (ref: Married)				
Divorced/Widowed	1.014 (0.715–1.438)	0.937	1.928 (1.479–2.515)	<0.0001
Single	0.670 (0.162–2.772)	0.5803	3.759 (0.909–15.540)	0.0675
Region (ref: Metropolitan)				
Urban	0.879 (0.659–1.173)	0.38	1.016 (0.768–1.344)	0.9129
Rural	1.109 (0.831–1.479)	0.4836	1.073 (0.806–1.430)	0.6294
Self-rated health (ref: Good)				
Moderate	1.657 (1.096–2.505)	0.0166	1.449 (0.882–2.381)	0.1431
Poor	3.132 (2.041–4.808)	<0.0001	2.197 (1.351–3.575)	0.0015
Social contact frequency (ref: Everyday)				
2–3 times/week	1.110 (0.786–1.569)	0.5529	1.021 (0.733–1.422)	0.9011
1–2 times/month	0.985 (0.663–1.463)	0.9396	1.207 (0.797–1.829)	0.3733
5–6 times/year	1.131 (0.687–1.861)	0.6286	1.370 (0.875–2.144)	0.1685
Rarely	1.581 (1.090–2.294)	0.0159	1.958 (1.389–2.759)	0.0001

**Note.** Physical activity and dementia were modeled separately in sex-stratified analyses. Reference groups were defined as inactive and no dementia, respectively.

## Data Availability

Restrictions apply to the availability of these data. The data were obtained from the Korean Longitudinal Study of Aging (KLoSA), conducted by the Korea Employment Information Service. Access to the data requires approval from the data provider, and the authors do not have permission to publicly share the raw data.

## Supplementary Materials

The following supporting information can be downloaded at: https://www.mdpi.com/article/10.3390/medicina62040628/s1, Table S1: Association between physical activity and all-cause mortality; Table S2: Association between dementia and all-cause mortality; Table S3: Association between Joint physical activity-dementia model and all-cause mortality; Figure S1: Kaplan-Meier curves for all-cause mortality according to joint categories of physical activity and dementia.
