# Supplementary material for: The Intersection of Physical Inactivity, Dementia, and Gender: A Population-Based Study of All-Cause Mortality in Middle-Aged and Older Adults in Korea
Source: Medicina (Kaunas). 2026 Mar 26;62(4):628. doi: 10.3390/medicina62040628 (PMC13117097; doi:10.3390/medicina62040628)
Supplement: Supplementary file 1 [file medicina-62-00628-s001.zip › medicina-4202089-supplementary.pdf]

Supplementary

Table S1. Association between physical activity and all-cause mortality

Variables	Adjusted HR (95% CI)	P-value
**Exercise level (ref: Inactive)**		
Sufficient Exercise (≥150)	0.714 (0.561–0.909)	0.006
**Depression (ref: No)**		
Yes	1.874 (1.423–2.467)	<.0001
**Gender (ref: Female)**		
Male	2.229 (1.836–2.705)	<.0001
**Age group (ref: ≤64 years)**		
65–74 years	2.159 (1.460–3.192)	<.0001
≥75 years	7.824 (5.403–11.325)	<.0001
**Education level (ref: ≥College)**		
≤Elementary school	1.467 (1.012–2.126)	0.045
Middle school	1.435 (0.960–2.147)	0.078
High school	1.145 (0.776–1.689)	0.497
**Marital status (ref: Married)**		
Divorced/Widowed	1.506 (1.243–1.825)	<.0001
Single	1.265 (0.464–3.449)	0.647
**Region (ref: Metropolitan)**		
Urban	0.959 (0.786–1.171)	0.345
Rural	1.103 (0.901–1.349)	0.201
**Self-rated health (ref: Good)**		
Moderate	1.540 (1.123–2.113)	0.008
Poor	2.748 (1.995–3.786)	<0.001
**Social contact frequency (ref: Everyday)**		
2–3 times/week	1.079 (0.850–1.368)	0.531
1–2 times/month	1.131 (0.855–1.497)	0.396
5–6 times/year	1.305 (0.936–1.820)	0.117
Rarely	1.909 (1.488–2.449)	<0.001

Table S2. Association between dementia and all-cause mortality

Variables	Adjusted HR (95% CI)	P-value
**Dementia (ref: No)**		
Yes	2.287 (1.651–3.169)	<0.001
**Depression (ref: No)**		
Yes	1.761 (1.332–2.328)	<0.001
**Gender (ref: Female)**		
Male	2.233 (1.839–2.709)	<0.001
**Age group (ref: ≤64 years)**		
65–74 years	2.094 (1.417–3.094)	<0.001
≥75 years	7.480 (5.169–10.822)	<0.001
**Education level (ref: ≥College)**		
≤Elementary school	1.548 (1.069–2.241)	0.0218
Middle school	1.485 (0.996–2.214)	0.0537
High school	1.172 (0.801–1.715)	0.4252
**Marital status (ref: Married)**		
Divorced/Widowed	1.560 (1.279–1.903)	<0.001
Single	1.426 (0.523–3.890)	0.488
**Region (ref: Metropolitan)**		
Urban	0.963 (0.789–1.177)	0.708
Rural	1.144 (0.962–1.361)	0.187
**Self-rated health (ref: Good)**		
Moderate	1.550 (1.133–2.121)	0.007
Poor	2.733 (1.993–3.749)	<0.001
**Social contact frequency (ref: Everyday)**		
2–3 times/week	1.091 (0.860–1.384)	0.473
1–2 times/month	1.140 (0.859–1.513)	0.366
5–6 times/year	1.299 (0.931–1.813)	0.123

Variables	Adjusted HR (95% CI)	P-value
Rarely	1.823 (1.417–2.346)	<0.001

Table S3. Association between Joint physical activity–dementia model and all-cause mortality

Variables	Adjusted HR (95% CI)	P-value
**Joint PA-dementia group (ref: Inactive + No dementia)**		
Sufficient Exercise + No dementia	0.722 (0.564–0.925)	0.01
Inactive + Dementia	2.302 (1.667–3.179)	<0.001
Sufficient Exercise + Dementia	1.050 (0.146–7.541)	0.962
**Depression (ref: No)**		
Yes	1.750 (1.321–2.318)	<0.001
**Gender (ref: Female)**		
Male	2.264 (1.854–2.765)	<0.001
**Age group (ref: ≤64 years)**		
65–74 years	2.376 (1.565–3.607)	<0.001
≥75 years	8.419 (5.698–12.440)	<0.001
**Education level (ref: ≥College)**		
≤Elementary school	1.452 (0.984–2.142)	0.061
Middle school	1.408 (0.927–2.139)	0.109
High school	1.193 (0.794–1.793)	0.396
**Marital status (ref: Married)**		
Divorced/Widowed	1.526 (1.250–1.864)	<0.001
Single	1.453 (0.533–3.958)	0.466
**Region (ref: Metropolitan)**		
Urban	0.950 (0.773–1.168)	0.627
Rural	1.102 (0.895–1.356)	0.362
**Self-rated health (ref: Good)**		
Moderate	1.480 (1.098–1.996)	0.018
Poor	2.530 (1.820–3.517)	<0.001
**Social contact frequency (ref: Everyday)**		
2–3 times/week	1.083 (0.846–1.386)	0.526
1–2 times/month	1.088 (0.808–1.463)	0.579
5–6 times/year	1.305 (0.932–1.828)	0.126
Rarely	1.769 (1.362–2.299)	<0.001

Note. Estimates for the "Sufficient exercise + Dementia" group should be interpreted with caution due to the small number of events.

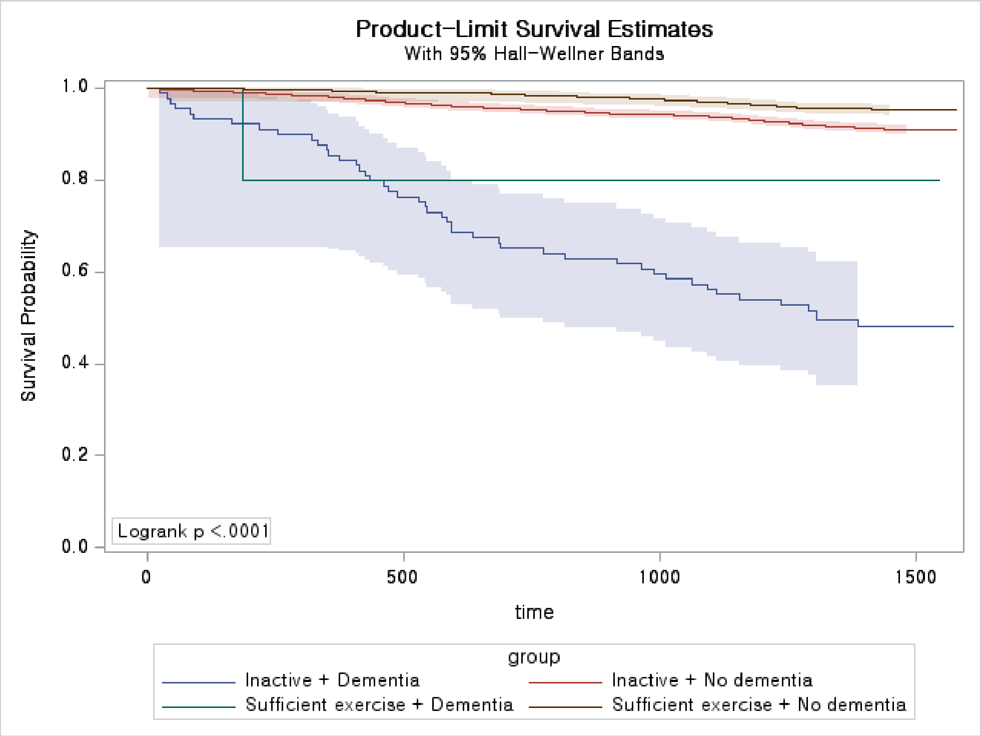

Figure S1. Kaplan–Meier curves for all-cause mortality according to joint categories of physical activity and dementia

Note. Participants were grouped based on physical activity level and dementia status. Estimates for some subgroups should be interpreted with caution due to small sample sizes.
